# Immune signatures of sepsis from mild infection to critical illness - a prospective observational study

**DOI:** 10.3389/fimmu.2025.1715812

**Published:** 2026-01-16

**Authors:** Timothy Arthur Chandos Snow, Francis Ryckaert, Ingrid Hass, Holly Pan, Samer Elkhodair, Mervyn Singer, David Brealey, Nishkantha Arulkumaran, The University College London Hospitals Critical Care Research Team, Naveed Saleem, Cesar Antonio, Alessia V. Waller, Deborah Smyth, Georgia Bercades, Alexandra Zapata Martinez, Laura Gallagher, Gladys Martir

**Affiliations:** 1 Bloomsbury Institute of Intensive Care Medicine, University College London, London, United Kingdom; 2 Emergency Department, University College London Hospital, London, United Kingdom; 3 UCLH NIHR Biomedical Research Centre, University College London Hospitals NHS Foundation Trust, London, United Kingdom

**Keywords:** immunotherapy, infections, lymphocytes, monocytes, sepsis

## Abstract

Sepsis-induced immunosuppression is a phenomenon characterized by the development of several changes in immunophenotype which predispose to secondary infections and increased mortality risk. Immunomodulatory therapies have yet to reproducibly demonstrate benefit in large clinical trials. We propose that several changes consistent with an immunosuppressive phenotype in sepsis represent either adaptive changes or epiphenomenon, rather than direct drivers of outcome in infection and sepsis. We therefore conducted a prospective observational study of patients presenting with infections with a spectrum of illness severity, to evaluate canonical features of monocyte and lymphocyte immunosuppression using flow cytometry. Several features consistent with immunosuppression in sepsis are observed in mild infections and non-infectious acute conditions. These features may be better understood as markers along a continuum of illness severity rather than distinct features of critical illness. Monocyte HLA-DR and co-stimulatory molecules (CD80 and CD86), and an increase in soluble PD-L1, discriminate between critically ill patients, patients with mild infection, and patients with non-infectious illness. In contrast, CD4^+^ and CD8^+^ lymphocyte phenotype did not discriminate between patient groups. Immunotherapies targeting lymphocyte function may only be effective if simultaneously augmenting monocyte antigen presentation and co-stimulatory pathways. Combination immunotherapy in sepsis requires evaluation.

## Introduction

Sepsis, the dysregulated host response to infection (1), is associated with substantial short- and long-term mortality. While many patients survive their initial presentation, a significant proportion do not survive to intensive care unit (ICU) or hospital discharge. Prolonged ICU stays are frequently complicated by persistent or secondary infections, often in the context of impaired immune cell function—commonly described as sepsis-induced immunosuppression (2).

Several immunological features have been linked to poor outcomes in sepsis. These alterations are typically more pronounced in patients who develop complications or ultimately die. Lymphopenia and reduced monocyte HLA-DR expression are among the most consistently reported phenotypes associated with sepsis-induced immunosuppression (3–6).

Despite over two decades of clinical trials, therapeutic strategies aimed at reversing immunosuppressive phenotypes have yet to be translated into routine practice. We hypothesize that many of the changes described as ‘sepsis-induced immunosuppression’ represent epiphenomena rather than direct drivers of outcome, which may explain the limited therapeutic progress.

**Figure 1:**
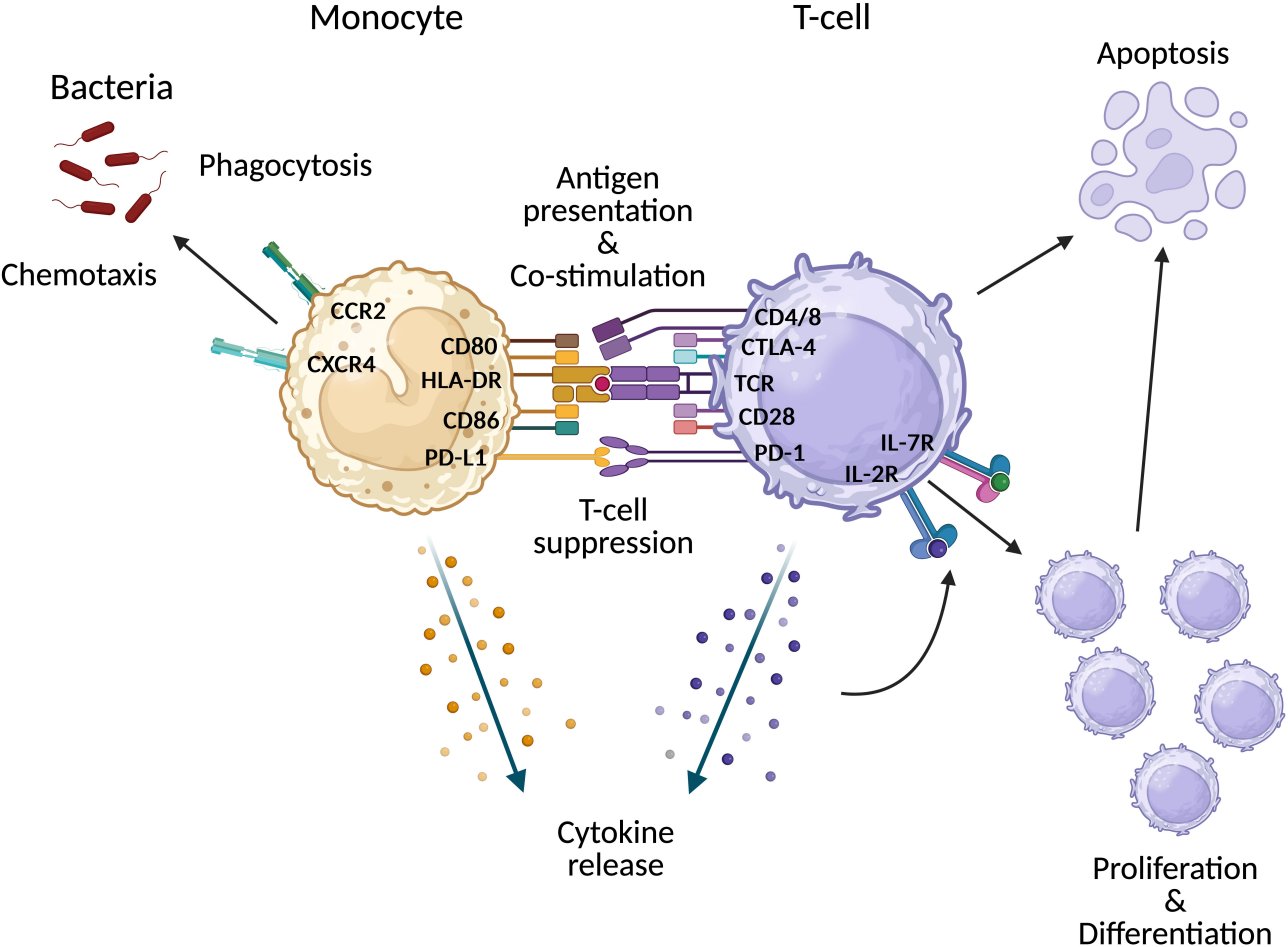
Summary of markers and associated functions assessed by immunophenotyping. CCR2, C-C motif chemokine receptor 2; CTLA-4, Cytotoxic T-lymphocyte associated protein-4; CXCR4, C-X-C motif chemokine receptor 4; CD, Cluster of differentiation; HLA-DR, Human leukocyte antigen – DR isotope; IL, Interleukin; PD-1, Programmed death receptor 1; PD-L1, Programmed death-ligand 1; R, Receptor. Created in BioRender.

We propose that such epiphenomena are better understood as markers along a continuum of illness severity rather than distinct features of critical illness, and that similar immune alterations may be observed in non-infectious acute conditions. To test this, we conducted a prospective observational study evaluating canonical features of monocyte and lymphocyte dysfunction in sepsis using flow cytometry, (**Figure 1**) in addition to functional assays (monocyte phagocytosis and LPS-induced cytokine release in whole blood) across the spectrum of infection severity—including patients with mild infection through to critical illness—and in patients with acute, non-infectious illness.

## Methods

### Ethical approval

Ethical approval was granted by the London – Queen Square Research Ethics Committee (REC reference 20/LO/1024) and the University College London Research Ethics Committee (REC reference 19181/001) for sample and data collection of patients and healthy volunteers respectively.

### Study design and participants

We performed a prospective observational cohort study recruiting patients aged ≥18 years presenting to the Emergency Department (ED) or admitted to the ICU at University College London Hospitals (UCLH) between 1^st^ Aug 2020 and 26^th^ January 2023 who had blood cultures taken for suspicion of bacterial infection. Exclusion criteria for this study included patients with severe anemia and a contra-indication to blood transfusion, those not expected to survive beyond 24 hours of admission (defined as palliative admission or to enable subsequent organ donation), haemato-oncological patients, patients on chronic immunosuppressive treatments, and pregnant women.

Samples from healthy volunteers were obtained from members of staff at University College London (UCL). Patient demographics, clinical data (physiology, diagnoses), laboratory data, and clinical outcomes were recorded from electronic healthcare records on a standardized data collection form. Patients were followed up to hospital discharge/death.

Based on our previous data in surgical patients (7) demonstrating a monocyte HLA-DR median fluorescence intensity (MFI) of 5,000 ± 1,250, with a power of 80%, and alpha of 0.05, a sample size of ≥ 25 patients per group were included to detect a statistically significant difference of 20% between groups.

### Sample collection and processing

Sample collection occurred at time of blood culture, blood was drawn into SST™ II Advanced Plus, K2 EDTA (Ethylenediamine tetra-acetic acid), and CPT™ vacutainers (all Becton Dickinson (BD) UK, Wokingham, UK) and processed within 1 hour of collection. SST™ II vacutainers were centrifuged at 1500g for 15minutes at room temperature and the serum aspirated and stored at -80°C.

500µl of EDTA blood was placed into an Eppendorf and LPS (lipopolysaccharide, Merck, Gillingham, UK) added to a final concentration of 100ng/ml. The dose was based on previously optimized protocols (8). Samples were incubated for 1 hour at 37°C, 5% CO_2_ before being centrifuged (5000g for 5 minutes) and the serum aspirated, frozen and stored at -80°C.

The CPT™ vacutainers were centrifuged at 1500g for 15 minutes at room temperature, the peripheral blood mononuclear cell (PBMC) layer transferred into Eppendorf reaction tubes, and washed once with phosphate buffered saline (PBS, Gibco, Thermo Fisher Scientific, Cambridge, UK) at 400g for 5 minutes at room temperature. Cells were resuspended in freezing media [fetal bovine serum (FBS, Thermo Fisher) with 10% dimethyl sulfoxide (DMSO, Thermo Fisher)], transferred to cryovials, placed in a Mr Frosty™ (Thermo Fisher) isoalcohol freezing chamber, and stored initially at -80°C before transfer into liquid nitrogen within 48 hours.

### Flow cytometry

Frozen PBMCs were defrosted in batches using PBS and counted using an automated cell counter (Countess 3, Thermo Fisher) prior to immediate staining.

For assessment of monocyte cell surface markers, PBMCs were centrifuged and resuspended in PBS with antibodies to the following cell surface markers (CD14, CD16, HLA-DR, CD80, CD86, CD184 (CXCR4), CD192 (CCR2), and CD274 (PD-L1), and viability stain (Aqua UV Live/Dead). After incubation for 30 minutes, samples were washed, resuspending in PBS, and placed on ice for subsequent analysis. Products and concentrations used are detailed in **Supplementary Table 1**.

To assess intracellular cytokines, PBMCs were suspended in PBS with antibodies to the following cell surface markers (CD14, CD16, and HLA-DR), and viability stain (Aqua UV Live/Dead). After incubation for 30 minutes, samples were washed and then underwent intracellular cytokine staining using the BD fixation/permeabilization kit as per manufacturer recommendations. The plate was centrifuged at 400g for 5 minutes, and the cells resuspended in fixation/permeabilization solution, incubated for 20 minutes at 4°C before being washed and resuspended in permeabilization/wash buffer with antibodies to the following intracellular cytokines (IL-1β, IL-10, and TNF-α). Following incubation for 30 minutes at 4°C, cells were washed and resuspended in PBS. Products and concentrations used are detailed in **Supplementary Table 1**.

For assessment of monocyte phagocytosis, PBMCs were resuspended in cell medium to a concentration of 1x10^6^ cells/ml and rested for 1 hour prior to stimulation. PBMCs were then plated on a 96-well plate at a concentration of 200,000 PBMCs per well with pHrodo red *E. coli* bioparticles (Thermo Fisher) to a final concentration of 100µg/ml and incubated at 37°C, 5% CO2 for 40 minutes. The dose was based on previously optimized protocols (8). Cells were then stained with antibodies to the following cell surface markers (CD14, CD16 and HLA-DR) and viability stain (Blue UV Live/Dead) and incubated for a further 30 minutes. Samples were then centrifuged resuspended in PBS and placed on ice. Products and concentrations used are detailed in **Supplementary Table 1**.

For assessment of lymphocyte cell surface markers, PBMCs were resuspended in annexin buffer and stained with antibodies to the following cell surface markers [CD3, CD4, CD8, CD19, CD25 (IL-2R), CD28, CD127 (IL-7R), CD152 (CTLA-4), and CD279 (PD-1)], viability stain (Aqua UV Live/Dead), and apoptosis stain (Annexin). After incubation for 30 minutes, samples were placed on ice in preparation for acquisition. Products and concentrations used are detailed in **Supplementary Table 2**.

**Figure 2:**
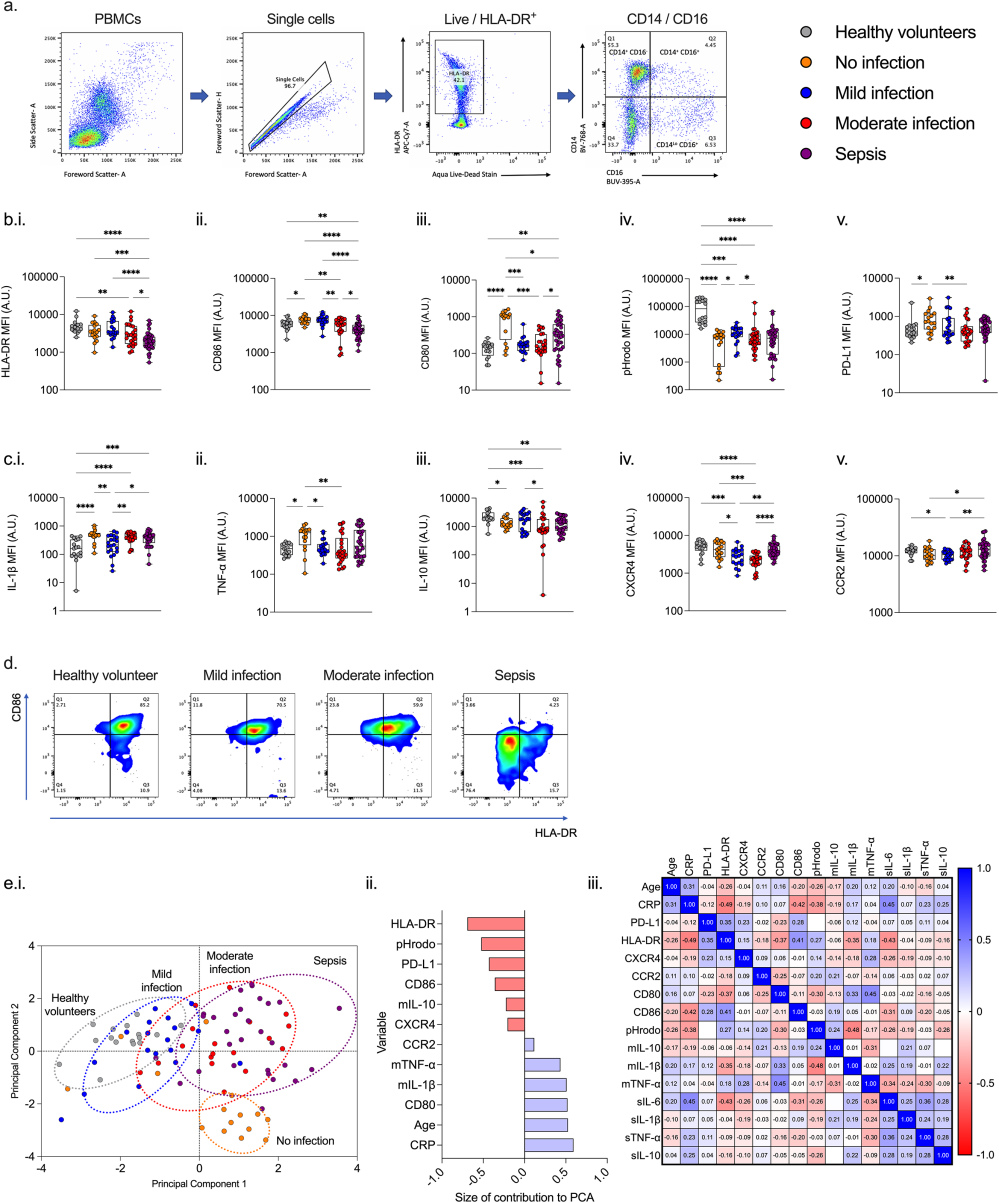
Monocyte immunophenotype changes with increasing severity of infection. **(a.)** Example gating strategy. Increasing infection severity is associated with alteration in immunophenotype characterized by expression of **(b.i.)** HLA-DR, **(b.ii.)** CD86, **(b.iii.)** CD80, **(b.iv.)** phagocytosis (measured using pHRodo bioparticles), **(b.v.)** PD-L1, **(c.i.)** IL-1b, **(c.ii.)** TNF-a, **(c.iii.)** IL-10, **(c.iv.)** CXCR4, and **(c.v.)** CCR2. **(d.)** As infection severity increases, there is a reduction in both costimulatory receptor CD86 and HLA-DR expression. **(e.i.)** Principal component analysis demonstrates that increasing severity of infection represents a continuum of immunophenotype whilst the no infection group demonstrates good separation between those with and without infection, **(e.ii.)** with HLA-DR and CD80 providing the best markers of separation. **(e.iii.)** Serum cytokines (prefaced with s) correlate poorly with monocyte immunophenotype including intracellular cytokines (prefaced by m). *p<0.05, **p<0.01, ***p<0.001, and ****p<0.0001.

**Figure 3:**
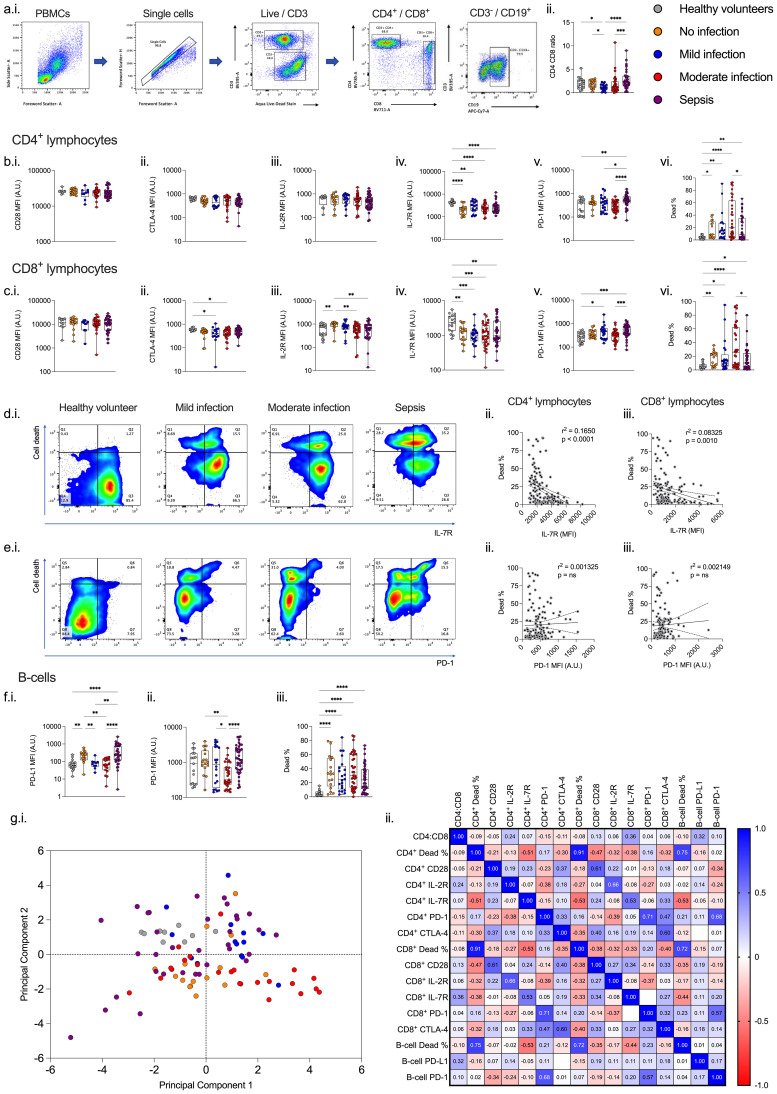
Lymphocyte immunophenotype changes with increasing severity of infection. **(a.i.)** Example gating strategy. Increasing infection severity is associated with alteration in immunophenotype characterized by changes in **(a.ii.)** CD4:CD8 and in **(b.)** CD4+ and **(c.)** CD8+ lymphocyte expression of **(i.)** CD28, **(ii.)** CTLA-4, **(iii.)** IL-2R, (iv.) IL-7R, **(v.)** PD-1, and **(vi.)** cell death. **(d.)** IL-7R expression correlates with cell death in both (i. and ii.) CD4+ and (iii.) CD8+ lymphocytes, whereas **(e.)** PD-1 expression does not correlate with cell death in (**i.** and **iii.**). Infection severity is associated with expression of **(f.)** B lymphocyte **(i.)** PD-L1, **(ii)** PD-1, and **(iii.)** cell death. **(g.i.)** Principal component analysis does not demonstrate separation between healthy volunteers and patients with increasing severity of infection or no infection. **(e.ii.)** Only cell death correlates between the lymphocyte subsets. *p<0.05, **p<0.01, ***p<0.001, and ****p<0.0001.

Cells were acquired on an LSR II flow cytometer (BD) running FACSDiva version 9 (BD). Calibrations beads (BD) were run prior to each experiment and compensation controls were applied to all samples prior to analysis. Single-stained unstimulated healthy donor cells were used as compensation controls for cell surface markers. Healthy donor cells were heat-treated for 10 minutes at 60°C as a positive control for viability stains. Compensation beads (BD) were used as positive controls for intracellular cytokines. FMO (fluorescence minus one) samples for all fluorophores were used to identify cell populations. Cell populations of interest were identified using the following Boolean gating strategy: lymphocytes or PBMCs, singlets, live cells, and cell surface markers and stopping gate set at 10,000 events for either CD14^++^CD16^-^ monocytes or CD4^+^ lymphocytes. An example gating strategy used to identify monocytes is shown in **Figure 2** and lymphocytes in **Figure 3**.

### Cytokine and chemokine measurements

Serum IL-1β, IL-6, IL-10, and TNF-α, soluble PD-1, and soluble PD-L1 levels were measured using Duoset ELISA kits (R&D Systems, Minneapolis, MN) as per manufacturer instructions. IL-1β, IL-6, IL-10, and TNF-α, were measured in serum from LPS-stimulated whole blood samples. Serum samples were diluted 1:2 in reagent dilutant. Optical densities were acquired on a SPECTROstar Nano microplate reader (BMG Labtech, Aylesbury, UK).

In a cohort of randomly selected 80 patients/volunteers, electrochemiluminescent immunoassays were performed according to the manufacturer’s instructions (Meso Scale Discovery (MSD), Rockville, MD). For analysis, two 10- inflammatory marker panels including IFN-γ, IL-4, IL-6, IL-8, IL-12p70, IL-5, GM-CSF, G-CSF, IFN-α2a, IFN-β, IL-1RA, IL-7, IL-19, IP-10, MCP-1, MIP-1α, and VEGF-A were used. Electrochemiluminescence was acquired using a Meso QuickPlex SQ120 microplate reader (MSD).

### Subgroup analysis

Additional *post-hoc* analysis was performed to assess immunotype in patients in the moderate infection and sepsis cohorts who had positive microbiological cultures compared to those who did not, and patients with a non-resolving (prolonged) infection defined as requiring an antibiotic course length of greater than 10 days compared to patients with an uncomplicated infection (9).

### Statistics

Analysis of clinical data were performed using anonymized data. Continuous and categorical variables are reported as median (interquartile range) and n (%), respectively. Mann Whitney U test was used for comparison of continuous variables between groups. Categorical data were compared using the chi-square test.

Flow cytometry data were analyzed using FlowJo (version 10.7.1, BD). Samples with cell counts fewer than 50 in the population of interest were excluded. Data are presented as either median fluorescence intensity (MFI; arbitrary units) or percentage positive cells with interquartile ranges. Multiplex data were analyzed using MSD Discovery Workbench (version 4.0, MSD) and ELISA data were analyzed using MARS (version 3.42, BMG). Both are presented as mean concentration with standard deviation. Differences between groups were compared using Kruskal-Wallis with Dunn’s uncorrected test for multiple comparisons. Graphs were constructed, and statistical analysis performed using Prism version 9 (GraphPad). Missing data is summarized in **Supplementary Table 3**.

## Results

### Clinical data

A total of 117 patients were included, 21 (18%) patients who attended the emergency department (ED) and were discharged home with a course of oral antibiotics (mild infection group), 37 (32%) patients who attended the ED with infections and were admitted to a general medical ward (moderate infection group), 42 (36%) critically ill patients with an infection on the ICU (sepsis group), and 17 (15%) patients who attended the ED with non-infectious acute conditions (no infection control group). Additionally, 17 healthy volunteers were included for reference. A diagram summarizing the cohorts is available in **Supplementary Figure 1** and demographic and clinical data are shown in **Supplementary Table 4**. Time from ICU admission to recruitment and first sampling was 3 (1–6) days. In-hospital mortality among ICU patients was 29% and no patients admitted from the ED to the ward died in hospital. Monocyte, lymphocyte, and neutrophil count were similar between patient cohorts.

### Monocyte phenotype

Monocyte HLA-DR and CD86 were lower in patients with sepsis compared to all other cohorts (p<0.05); with an association between increasing illness severity and lower levels of expression. (**Figures 2.b.i., b.ii., c**.) Patients with moderate infection had lower HLA-DR expression compared to healthy volunteers (p<0.01), but were similar to levels seen in patients with mild infections and no infection. (**Figure 2.b.i.**) In contrast, expression of CD86 were lower among patients with moderate infections compared to patients with mild infections and those with no infection (p<0.01). (**Figure 2.b.ii**.) Expression of CD80 was higher among patients with no infection compared to all other patient cohorts (p<0.05). (**Figure 2.b.iii**.) There were no consistent differences between increasing severity of illness among cohorts of patients and changes in monocyte PD-L1, or chemotaxis markers, intracellular cytokines, and phagocytosis. (**Figures 2.b.iv.-v, c.i.-v**., **Supplementary Figure 2**).

Principal component analysis (PCA) was conducted in 96 individuals for whom full datasets were available for 12 monocyte variables, CRP, and age. PCA demonstrated separation between the sepsis, no infection, and healthy volunteer groups. (**Figure 2.e.i**.) The first two principal components provided 38% cumulative proportion of variance, with monocyte HLA-DR (coefficient of 0.73), followed by co-stimulatory molecule CD80 providing the greatest discrimination between patients. (**Figure 2.e.ii**.) A significant inverse correlation was seen between monocyte HLA-DR expression and CRP (r=-0.49) and CD80 (r=-0.37). Levels of serum cytokines and monocyte intracellular cytokines IL-1β, IL-10 and TNF-α did not correlate. (**Figure 2.e.iii**.).

### Lymphocyte phenotype

Among CD4^+^ lymphocytes, IL-7R expression was lower and cell death higher among all patient cohorts compared to healthy volunteers (p<0.05), with no differences in IL-7R expression between patient cohorts. (**Figures 3.b.iv., b.vi., d.i. and e.i**.) A similar pattern was seen with CD8^+^ lymphocytes (p<0.05). (**Figures 3.c.iv., c.vi**.) PD-1 expression was higher among sepsis patients compared to patients with moderate infections and healthy volunteers (p<0.01). (**Figures 3.b.v., c.v**.) However, there was no clear association between illness severity and levels of PD-1 expression. We did not find any association between CD28 (**Figures 3.b.i., c.i**.) or IL-2R (**Figures 3.b.iii., c.iii**.) expression and illness severity. Levels of CD8^+^ lymphocyte CTLA-4 were lower among patients with mild and moderate infections compared to healthy volunteers (p<0.05), however there were no differences in CD4^+^ lymphocytes. (**Figures 3.b.ii., c.ii**., and **Supplementary Figure 3**).

There was a weak (albeit statistically significant) inverse correlation between IL-7R expression and cell death in CD4^+^ (r^2^ = 0.1650, p<0.0001) and CD8^+^ (r^2^ = 0.08325, p=0.0010) lymphocytes. (**Figures 3.d.ii, d.iii**.) We found no robust correlation between PD-1 expression and cell death in CD4^+^ and CD8^+^ lymphocytes. (**Figures 3.e.ii, e.iii**).

Among B lymphocytes, cell death was higher among all patient cohorts compared to healthy volunteers (p<0.001); but no differences between patient cohorts. (**Figure 3.f.iii**.) Expression of PD-1 and PD-L1 were not associated with increasing illness severity. (**Figures 3.f.i., f.ii**).

Lymphocyte markers did not discriminate between patient cohorts on principle component analysis and only cell death correlated between the lymphocyte subsets (**Figures 3.g.i, g.ii**.)

### Cytokines

**Figure 4:**
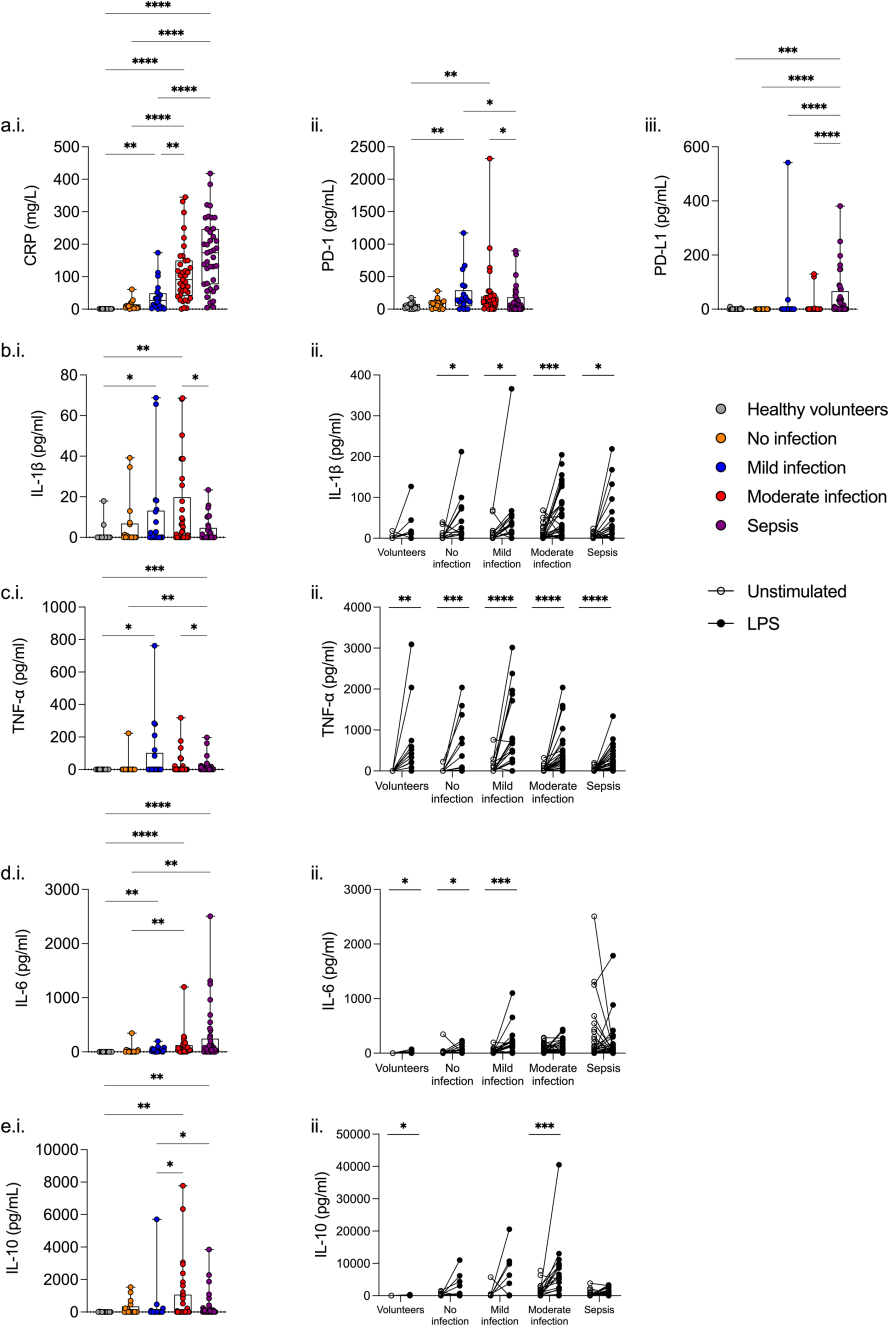
Changes in serum biomarkers with increasing severity of infection. Serum levels of **(a.i.)** CRP, **(a.ii.)** PD-1, **(a.iii.)** PD-L1, **(b.i.)** IL-1β, **(c.i.)** TNF-α, **(d.i.)** IL-6, and **(e.i.)** IL-10 were measured and compared between groups of infection. Additionally, cell anergy was assessed in a whole blood lipopolysaccharide (LPS) model comparing **(b.ii.)** IL-1β, **(c.ii.)** TNF-α, **(d.ii.)** IL-6, and **(e.ii.)** IL-10 before (white dot) and after (black dot) LPS-stimulation. *p<0.05, **p<0.01, ***p<0.001, and ****p<0.0001.

Serum levels of soluble PD-L1 but not PD-1 were significantly higher among sepsis patients compared to all other patient groups (p<0.001), although did not distinguish between other patient cohorts and healthy volunteers. (**Figure 4.a.ii., a.iii**.) Serum CRP and IL-6 increased with illness severity; levels being higher among sepsis patients compared to all other patient cohorts and healthy volunteers (p<0.01). (**Figures 4.a.i., d.i**.) Additionally, levels of CRP and IL-6 were higher among patients with infections but not statistically different compared to patients with no infection. (**Figures 4.a.i., d.i**.).

All patient cohorts and healthy volunteers had a significant increase in TNF-α on *ex vivo* LPS stimulation (p<0.01). (**Figure 4.c.ii**.) However, healthy volunteers did not demonstrate an increase in IL-1β, whereas all patient groups did (p<0.05). (**Figure 4.b.ii**.) Sepsis patients and patients with moderate infection did not have a significant increase in IL-6 on *ex vivo* LPS stimulation, whereas all other cohorts did (p<0.05). (**Figure 4.d.ii**.) There was no clear association between IL-10 release and illness severity. (**Figure 4.e.ii**.).

Among the exploratory panel of cytokines tested on multiplex, there were no cytokines that reliably distinguished between sepsis patients and other patient cohorts; nor patients with no infection and acute infection (**Supplementary Figure 4**).

### Subgroups

An exploratory analysis was performed to assess the effect of having a positive microbiological culture in the moderate infection and sepsis cohorts. A causative organism was identified on culture in 19 (51%) of patients with moderate infection and 74% of patients with sepsis. (**Supplementary Table 5**) There were no statistically significant differences between immune markers between patients with or without positive cultures. (**Supplementary Figure 5**).

An additional analysis assessed the effect of non-resolving or recurrent infection in the moderate infection and sepsis cohorts on immunophenotype. Non-resolving or recurrent infection was diagnosed in 18 (49%) of patients with moderate infections and 29 (69%) of sepsis patients. (**Supplementary Table 5**) Among the immune markers assessed, there were no discriminating features between patients with or without prolonged or recurrent infections. (**Supplementary Figure 6**).

## Discussion

We confirm previous findings, strengthening the external validity of our data, while also presenting several novel observations. Multiple features consistent with immunosuppression were prevalent among critically ill patients with sepsis. This included lower monocyte HLA-DR and CD86 expression, elevated CD80 expression, increased lymphocyte PD-1 expression, increased cell death, and reduced lymphocyte IL-7 receptor (IL-7R) expression.

Univariate analysis and PCA highlighted monocyte HLA-DR and its co-stimulatory molecules CD80 and CD86 providing discriminatory value between critically ill patients or patients with non-infectious illness and patients with infection and sepsis. CD4^+^ and CD8^+^ lymphocyte phenotype, however, did not discriminate between critically ill patients or patients with non-infectious illness and patients with infection and sepsis. Among the soluble mediators assessed, PD-L1 levels were significantly higher among critically ill patients compared to all other patient groups, and CRP demonstrated a positive correlation with disease severity.

A reduction in monocyte HLA-DR is one of the most robust features associated with poor outcomes in sepsis, including secondary infections and mortality (6). Reduced expression of monocyte HLA-DR is thus regarded as a marker of immunosuppression in sepsis. We found the reduction in monocyte HLA-DR (and associated changes to monocyte phenotype) in patients admitted to the ICU also occur in mild uncomplicated infections; albeit to a lesser degree, representing a continuum of illness severity rather than a distinctive change in sepsis.

Several factors are known to regulate the expression of monocyte HLA-DR in sepsis, including activity of the Class II transactivator (CIITA) (10), glucocorticoids (via the suppression of CIITA transcription) (11), and IL-10 (via endocytosis, resulting in intracellular sequestration) (12, 13). Expression of healthy volunteer HLA-DR is reduced by circulating inflammatory mediators (12, 13).

The reduction in HLA-DR with associated increase in CD80 and concurrent reduction in CD86 expression has been previously associated with higher illness severity in sepsis (14). CD86 is an important target for immune regulation and control of T lymphocyte CD28 co-stimulation, while CD80 may attenuate lymphocyte CTLA-4 function through altered trafficking (15). Together, the reduction in monocyte CD86 and HLA-DR suggest that the monocyte phenotype in sepsis may not trigger effector cell function of T lymphocytes in sepsis. Therapeutic strategies, including IFN-γ upregulate monocyte CD80, CD86, and HLA-DR (16). However, immunomodulatory therapies targeting monocyte function have yet to reproducibly demonstrate benefit in large clinical trials (17, 18).

Lymphocytes demonstrated elevated levels of PD-1 among critically ill patients; albeit with no clear association with cell death. Although monocyte PD-L1 expression was not significantly elevated, illness severity-dependent increase in B lymphocyte PD-L1 expression was seen. Activation of the PD-1/PD-L1 axis may result in T lymphocyte anergy in sepsis. Increased PD-1/PD-L1 axis and CTLA-4 expression on lymphocytes is associated with lymphocyte anergy and mortality among patients who die from sepsis (19). The significant proportion of B lymphocyte death (20) and increased proportion in PD-L1^+^ B lymphocytes in sepsis (21) has also been described by others.

Expression of lymphocyte IL-7R was not associated with illness severity, nor did it discriminate between patients with acute non-infectious illness and patients with infection. However, expression of IL-7R was lower and cell death higher among all patient cohorts compared to healthy volunteers, with significant association between IL-7R and cell death. Therapeutic interventions including PD-1 receptor antagonists and recombinant IL-7 are at the early stages of clinical investigation (22–25). Notably, in contrast to prior reports (3, 19, 26), we observed no differences in lymphocyte CD28 expression and reduced CTLA-4 levels in sepsis patients. However, a reduction in splenic CD4^+^ lymphocyte CTLA-4 MFI was previously reported (3).

C-reactive protein (CRP), a prototypical marker of illness severity in inflammatory conditions, is routinely measured in many ICUs. CRP demonstrated a positive association with disease severity. IL-6, a key regulator of CRP production, demonstrated a similar pattern. The discriminative ability of CRP to distinguish patients of different illness severity out-performed a panel of 20 other serum biomarkers. We found no correlation between serum and monocyte intracellular TNF-α, IL-1β, or IL-10 despite monocytes being a rich source of cytokines IL-1β, TNF-α, and IL-10 (27).

Serum levels of soluble PD-L1 were significantly higher among critically ill patients compared to all other patient groups, although did not distinguish between other patient cohorts and healthy volunteers. Others have also described elevated levels of soluble PD-L1 in sepsis compared to other inflammatory conditions (28). Elevated levels of serum PD-L1 may contribute to T lymphocyte anergy in sepsis. The utility of serum PD-L1 as a theragnostic biomarker for therapy with PD-1/PD-L1 pathway blockade in sepsis requires investigation.

While the expression of cell surface receptors is often used as a proxy for immune cell activation or function, the dynamic response of immune cells to stimuli (e.g., LPS) is particularly relevant in the context of secondary infections. The use of *ex vivo* LPS-induced cytokine release in whole blood has thus been proposed as a surrogate for dynamic immune function in sepsis. However, data on LPS-induced cytokine production are conflicting, which may be due to the differences in duration of incubation – ranging from 30 minutes to 24 hours (29–33). We chose to perform a 1-hour model as this represented the most pragmatic opportunity for translational potential as a ‘bed-side’ test. However, we found that LPS-induced *ex vivo* cytokine release did not reliably discriminate critically ill sepsis patients from other cohorts; nor did it discriminate patients with non-infectious acute illness from other patient cohorts.

Despite the breadth of data presented, we acknowledge limitations. We report results from a single-center observational study and excluded some groups of patients including those with haemato-oncological malignancy which may limit the generalizability of our findings (8). Recruitment occurred during the later parts of the COVID-19 pandemic therefore we are unable to account for the effect of previous viral or vaccine exposure. Whilst there was an age difference between the groups, with volunteers and those with mild infection being young than other groups, we specifically included a no infection control group to account for this. We did not report differences between ICU survivors and non-survivors due to the relatively small sample size, and reported immunophenotype assessed at a single time-point. We may have therefore missed any dynamic changes to immunophenotype which distinguish between survivors and non-survivors (34). Performing sequential measurements to assess the trajectory of immune cell function could provide further information. We have also not quantified monocyte HLA-DR in terms of receptors per cell, which would require the use of flow cytometry within hours of blood sampling. Different patient populations were not age-matched, reflecting clinical data.

Data on intermediate and non-classical monocyte subsets are not presented as cell counts from patients were limited. However, quantification of cell surface markers on monocyte subsets are rarely, if ever, used to stratify immune status in critically ill patients. We assessed levels of ligands and receptors (e.g., PD-L1) on flow cytometry but were unable to assess their functional relevance or associated pathways.

Several studies have assessed the transcriptomic profile of immune cells; however, these may not reflect cell surface protein/receptor expression. Bulk transcriptomics also cannot directly assess the phenotype of specific cell subsets. The use of flow cytometry allows assessment of single cell protein expression. Using complementary methodologies and an iterative approach, we provide novel insights into immune signatures in patients with a range of illness severity and disease stages, and the use of *ex vivo* dynamic functional assays.

In summary, several features consistent with immunosuppression in sepsis are observed in mild infections and non-infectious acute conditions. These features may be better understood as markers along a continuum of illness severity rather than distinct features of critical illness. Reduced lymphocyte IL-7R and viability did not discriminate between ICU patients or patients with non-infectious acute illness from other patient cohorts. Monocyte antigen presentation and co-stimulatory pathway (reduced HLA-DR, CD86), and an increase in soluble PD-L1 discriminated ICU patients from other patient cohorts. Immunotherapies targeting lymphocyte function may only be effective if simultaneously augmenting monocyte antigen presentation and co-stimulatory pathways, therefore combination immunotherapy for sepsis requires further evaluation. The administration of immunotherapies to all patients with sepsis irrespective of their underlying immunophenotype, may be detrimental. Indeed, trials administering IFN-γ (18) and recombinant IL-7 (25) to augment monocyte and lymphocyte function respectively, has been associated with increased adverse outcomes. The possibility of targeted immunotherapy based on individual patient immunophenotype has recently been demonstrated in the ImmunoSep trial (35).

## Data Availability

The raw data supporting the conclusions of this article will be made available by the authors, without undue reservation.

## Group members of UCLH Critical Care Research Team

Naveed Saleem, Antonio Cesar, Alessia V. Waller, Deborah Smyth, Georgia Bercades, Alexandra Zapata Martinez, Laura Gallagher, and Gladys Martir.
