# Supplementary material for: Immune signatures of sepsis from mild infection to critical illness - a prospective observational study
Source: Front Immunol. 2026 Jan 16;16:1715812. doi: 10.3389/fimmu.2025.1715812 (PMC12855556; doi:10.3389/fimmu.2025.1715812)
Supplement: Supplementary file 1 [file Table1.docx]

Supplemental Material

# Supplemental Tables

| Cell Marker | Cell Function | Fluorochrome | Species/Isotype | Catalogue no. | Concentration |
| --- | --- | --- | --- | --- | --- |
| CD14 | Gating | BV785 | Mouse IgG2a | BL 301840 | 1:250 |
| CD16 |  | BUV395 | Mouse IgG1 | BD 563785 | 1:250 |
| HLA-DR | Antigen presentation | APC-Cy7 | Mouse IgG2a | BL 307618 | 1:250 |
| CD80 | Co-stimulation | PE | Mouse IgG1 | BL 305208 | 1:250 |
| CD86 |  | PE-Dazzle | Mouse IgG1 | BL 374218 | 1:250 |
| CD184 (CXCR4) | Chemokine receptor | BV421 | Mouse IgG2a | BL 306518 | 1:250 |
| CD192 (CCR2) |  | BV711 | Mouse IgG2a | BL 357232 | 1:250 |
| CD274 (PD-L1) | T-cell suppression | APC | Mouse IgG1 | BD 563741 | 1:250 |
| L/D Blue | Viability | Blue UV | - | TF L34962 | 1:1000 |
| pHrodo E. *coli* | Phagocytosis | PE | - | TF P35361 | 100mcg/mL |
| IL-1β | Cytokine | FITC | Mouse IgG1 | TF 11701842 | 1:100 |
| IL-6 |  | APC | Rat IgG1 | BD 561441 | 1:100 |
| IL-10 |  | BV421 | Rat IgG1 | BL 501422 | 1:100 |
| TNF-α |  | PE- Cy7 | Mouse IgG1 | BL 502930 | 1:100 |

Supplemental Table 1: Monocyte immunophenotype flow cytometry fluorochromes.

Abbreviations: APC: Allophycocyanin, BD: Beckton Dickinson, BL: Biolegend, BUV: Brilliant ultraviolet, BV: Brilliant violet, CCR2: C-C motif chemokine receptor 2, CXCR4: C-X-C motif chemokine receptor 4, CD: Cluster of differentiation, Cy: Cyanine, FITC: Fluorescein isothiocyanate, HLA-DR: Human leukocyte antigen – DR isotope, IL: Interleukin, L/D: Live/Dead, PD-L1: Programmed death-ligand 1, PE: Phycoerythrin, TF: Thermo Fisher, UV: Ultraviolet.

| Cell Marker | Cell Function | Fluorochrome | Cat no | Isotype | Dilution |
| --- | --- | --- | --- | --- | --- |
| CD3 | Gating | BUV395 | BD 564001 | Mouse IgG1 | 1:250 |
| CD4 |  | BV785 | BL 317442 | Mouse IgG2b | 1:250 |
| CD8 |  | BV711 | BD 563677 | Mouse IgG1 | 1:250 |
| CD19 |  | APC-Cy7 | BD 557791 | Mouse IgG1 | 1:250 |
| CD25 (IL-2R) | Differentiation | BV421 | BD 562442 | Mouse IgG1 | 1:250 |
| CD28 | Activation | BUV737 | BD 748475 | Mouse IgG1 | 1:250 |
| CD127 (IL-7R) | Proliferation | PE | BL 351304 | Mouse IgG1 | 1:250 |
| CD152 (CTLA-4) | Suppression | PE-Dazzle | BL 349922 | Mouse IgG1 | 1:250 |
| CD274 (PD-L1) |  | APC | BD 563741 | Mouse IgG1 | 1:250 |
| CD279 (PD-1) | Anergy | PE-Cy7 | BD 561272 | Mouse IgG1 | 1:250 |
| Annexin | Viability | Alexa Fluro 350 | TF A23202 | - | 1:250 |
| L/D Aqua |  | Aqua UV | TF L34957 | - | 1:1000 |

Supplemental Table 2: Lymphocyte flow cytometry fluorochromes.

Abbreviations: APC: Allophycocyanin, BD: Beckton Dickinson, BL: Biolegend, BUV: Brilliant ultraviolet, BV: Brilliant violet, CD: cluster of differentiation, CTLA-4: Cytotoxic T-lymphocyte associated protein-4, Cy: Cyanine, IL: Interleukin, L/D: Live/Dead, PD-1: Programmed death receptor 1, PD-L1: Programmed death-ligand 1, PE: Phycoerythrin, TF: Thermo Fisher, UV: Ultraviolet.

| **ID** | **Demographics (7)** | **Clinical Parameters (5)** | **Laboratory Values (10)** | **Serum cytokines (6)** | **Serum LPS Stimulation (4)** | **Lymphocyte apoptosis (10)** | **Lymphocyte immunophenotyping (36)** | **Monocyte immunophenotyping (14)** | **Monocyte phagocytosis (2)** | **Monocyte intracellular cytokines (4)** | **Serum Multiplex (20)** |
| --- | --- | --- | --- | --- | --- | --- | --- | --- | --- | --- | --- |
| HV-001 | 0 | 5 | 9 | 0 | 0 | 0 | 12 | 0 | 0 | 0 | 20 |
| HV-002 | 0 | 5 | 9 | 0 | 0 | 0 | 12 | 0 | 0 | 0 | 20 |
| HV-003 | 0 | 5 | 9 | 4 | 4 | 0 | 12 | 0 | 0 | 0 | 20 |
| HV-004 | 0 | 5 | 9 | 4 | 4 | 0 | 12 | 0 | 0 | 0 | 20 |
| HV-005 | 0 | 5 | 9 | 0 | 0 | 0 | 12 | 0 | 0 | 0 | 20 |
| HV-006 | 0 | 5 | 9 | 0 | 0 | 0 | 12 | 0 | 2 | 0 | 20 |
| HV-007 | 0 | 5 | 9 | 0 | 0 | 0 | 12 | 0 | 0 | 0 | 20 |
| HV-008 | 0 | 5 | 9 | 4 | 4 | 0 | 12 | 0 | 0 | 0 | 20 |
| HV-009 | 0 | 5 | 9 | 0 | 0 | 0 | 0 | 0 | 0 | 0 | 0 |
| HV-010 | 0 | 5 | 9 | 0 | 0 | 0 | 0 | 0 | 0 | 0 | 0 |
| HV-011 | 0 | 5 | 9 | 0 | 0 | 0 | 0 | 0 | 0 | 0 | 0 |
| HV-012 | 0 | 5 | 9 | 0 | 0 | 0 | 0 | 0 | 0 | 0 | 0 |
| HV-013 | 0 | 5 | 9 | 0 | 0 | 0 | 0 | 0 | 0 | 0 | 0 |
| HV-014 | 0 | 5 | 9 | 0 | 0 | 0 | 0 | 0 | 0 | 0 | 20 |
| HV-015 | 0 | 5 | 9 | 0 | 0 | 0 | 0 | 0 | 0 | 0 | 0 |
| HV-016 | 0 | 5 | 9 | 0 | 0 | 0 | 0 | 0 | 0 | 0 | 0 |
| HV-017 | 0 | 5 | 9 | 0 | 0 | 0 | 0 | 0 | 0 | 0 | 0 |
| ED-010 | 0 | 0 | 1 | 0 | 0 | 0 | 12 | 0 | 0 | 4 | 20 |
| ED-038 | 0 | 0 | 0 | 0 | 0 | 0 | 0 | 0 | 0 | 1 | 0 |
| ED-050 | 0 | 0 | 0 | 0 | 0 | 0 | 0 | 0 | 0 | 0 | 0 |
| ED-052 | 0 | 0 | 0 | 0 | 0 | 0 | 12 | 0 | 0 | 0 | 0 |
| ED-084 | 0 | 0 | 1 | 0 | 0 | 0 | 0 | 0 | 0 | 0 | 0 |
| ED-086 | 0 | 0 | 0 | 0 | 0 | 0 | 0 | 0 | 0 | 0 | 20 |
| ED-089 | 0 | 0 | 0 | 0 | 0 | 0 | 0 | 0 | 0 | 0 | 20 |
| ED-101 | 0 | 0 | 0 | 0 | 0 | 0 | 0 | 0 | 0 | 0 | 20 |
| ED-111 | 0 | 0 | 0 | 0 | 0 | 0 | 0 | 0 | 0 | 0 | 20 |
| ED-134 | 0 | 0 | 0 | 0 | 0 | 0 | 0 | 0 | 0 | 0 | 0 |
| ED-144 | 0 | 0 | 1 | 0 | 0 | 0 | 0 | 0 | 0 | 0 | 20 |
| ED-153 | 0 | 0 | 0 | 0 | 0 | 0 | 0 | 0 | 0 | 0 | 20 |
| ED-164 | 0 | 0 | 0 | 0 | 0 | 0 | 0 | 0 | 0 | 0 | 20 |
| ED-165 | 0 | 0 | 0 | 0 | 0 | 0 | 0 | 0 | 0 | 0 | 20 |
| ED-174 | 0 | 0 | 1 | 0 | 0 | 0 | 0 | 0 | 0 | 0 | 20 |
| ED-178 | 0 | 0 | 0 | 0 | 0 | 0 | 0 | 0 | 0 | 0 | 20 |
| ED-194 | 0 | 0 | 0 | 0 | 0 | 0 | 0 | 0 | 0 | 0 | 20 |
| ED-004 | 0 | 0 | 1 | 0 | 4 | 0 | 12 | 0 | 0 | 0 | 20 |
| ED-006 | 0 | 0 | 0 | 0 | 0 | 0 | 12 | 0 | 2 | 0 | 20 |
| ED-009 | 0 | 0 | 0 | 0 | 0 | 0 | 12 | 11 | 2 | 0 | 20 |
| ED-016 | 0 | 0 | 1 | 0 | 0 | 0 | 12 | 0 | 0 | 0 | 0 |
| ED-018 | 0 | 0 | 0 | 0 | 0 | 0 | 12 | 0 | 0 | 0 | 20 |
| ED-021 | 0 | 0 | 0 | 0 | 0 | 0 | 12 | 0 | 0 | 0 | 20 |
| ED-026 | 0 | 0 | 0 | 0 | 0 | 0 | 0 | 0 | 0 | 0 | 0 |
| ED-029 | 0 | 0 | 0 | 0 | 0 | 0 | 6 | 0 | 0 | 0 | 20 |
| ED-039 | 0 | 0 | 0 | 0 | 0 | 0 | 6 | 0 | 0 | 0 | 0 |
| ED-062 | 0 | 0 | 0 | 0 | 0 | 0 | 0 | 0 | 0 | 0 | 0 |
| ED-078 | 0 | 0 | 1 | 0 | 0 | 0 | 0 | 11 | 0 | 0 | 0 |
| ED-083 | 0 | 0 | 0 | 0 | 0 | 0 | 0 | 0 | 0 | 0 | 0 |
| ED-096 | 0 | 0 | 0 | 0 | 0 | 0 | 0 | 0 | 0 | 0 | 0 |
| ED-099 | 0 | 0 | 0 | 0 | 0 | 0 | 0 | 0 | 0 | 0 | 0 |
| ED-100 | 0 | 0 | 0 | 0 | 0 | 0 | 0 | 0 | 0 | 0 | 0 |
| ED-106 | 0 | 0 | 0 | 0 | 0 | 0 | 0 | 0 | 0 | 0 | 0 |
| ED-107 | 0 | 0 | 0 | 0 | 0 | 0 | 0 | 0 | 0 | 0 | 0 |
| ED-113 | 0 | 0 | 0 | 0 | 0 | 0 | 0 | 0 | 0 | 0 | 0 |
| ED-114 | 0 | 0 | 0 | 0 | 0 | 0 | 0 | 0 | 0 | 0 | 0 |
| ED-118 | 0 | 0 | 0 | 0 | 0 | 0 | 0 | 0 | 0 | 0 | 20 |
| ED-119 | 0 | 0 | 0 | 0 | 0 | 0 | 0 | 0 | 0 | 0 | 0 |
| ED-158 | 0 | 0 | 0 | 0 | 0 | 0 | 0 | 11 | 2 | 4 | 20 |
| ED-003 | 0 | 0 | 0 | 0 | 4 | 0 | 12 | 0 | 0 | 0 | 20 |
| ED-008 | 0 | 0 | 0 | 0 | 0 | 0 | 12 | 0 | 0 | 4 | 20 |
| ED-012 | 0 | 0 | 0 | 0 | 0 | 0 | 12 | 0 | 2 | 4 | 20 |
| ED-013 | 0 | 0 | 0 | 0 | 4 | 0 | 20 | 11 | 2 | 4 | 20 |
| ED-015 | 0 | 0 | 0 | 0 | 0 | 0 | 12 | 0 | 0 | 0 | 0 |
| ED-019 | 0 | 0 | 0 | 0 | 0 | 0 | 12 | 0 | 0 | 0 | 20 |
| ED-022 | 0 | 0 | 0 | 0 | 0 | 0 | 12 | 0 | 2 | 4 | 20 |
| ED-024 | 0 | 0 | 0 | 4 | 0 | 0 | 0 | 0 | 0 | 0 | 0 |
| ED-025 | 0 | 0 | 0 | 0 | 0 | 0 | 0 | 0 | 0 | 0 | 0 |
| ED-028 | 0 | 0 | 0 | 0 | 0 | 0 | 0 | 11 | 2 | 4 | 0 |
| ED-030 | 0 | 0 | 1 | 0 | 0 | 6 | 24 | 0 | 0 | 0 | 0 |
| ED-031 | 0 | 0 | 0 | 0 | 0 | 0 | 0 | 0 | 0 | 0 | 0 |
| ED-032 | 0 | 0 | 0 | 0 | 0 | 0 | 0 | 0 | 0 | 0 | 0 |
| ED-034 | 0 | 0 | 1 | 0 | 0 | 0 | 0 | 0 | 0 | 0 | 0 |
| ED-036 | 0 | 0 | 0 | 0 | 4 | 0 | 0 | 0 | 0 | 1 | 0 |
| ED-037 | 0 | 0 | 0 | 0 | 0 | 0 | 36 | 0 | 0 | 1 | 0 |
| ED-040 | 0 | 0 | 1 | 0 | 0 | 0 | 0 | 0 | 0 | 0 | 0 |
| ED-043 | 0 | 0 | 0 | 0 | 4 | 0 | 0 | 11 | 0 | 4 | 0 |
| ED-046 | 0 | 0 | 0 | 0 | 0 | 0 | 0 | 0 | 0 | 0 | 0 |
| ED-047 | 0 | 0 | 0 | 0 | 0 | 0 | 12 | 0 | 0 | 0 | 0 |
| ED-076 | 0 | 0 | 0 | 0 | 0 | 0 | 0 | 0 | 0 | 2 | 20 |
| ED-082 | 0 | 0 | 0 | 0 | 0 | 0 | 0 | 11 | 0 | 0 | 20 |
| ED-094 | 0 | 0 | 2 | 0 | 0 | 0 | 0 | 0 | 0 | 0 | 20 |
| ED-097 | 0 | 0 | 1 | 0 | 0 | 0 | 0 | 11 | 0 | 0 | 20 |
| ED-104 | 0 | 0 | 0 | 0 | 0 | 0 | 0 | 0 | 0 | 0 | 20 |
| ED-105 | 0 | 0 | 0 | 0 | 0 | 0 | 0 | 11 | 2 | 0 | 20 |
| ED-110 | 0 | 0 | 0 | 0 | 0 | 0 | 0 | 0 | 0 | 0 | 20 |
| ED-112 | 0 | 0 | 0 | 0 | 0 | 0 | 6 | 11 | 0 | 0 | 20 |
| ED-117 | 0 | 0 | 0 | 0 | 0 | 0 | 0 | 0 | 0 | 0 | 20 |
| ED-122 | 0 | 0 | 0 | 0 | 0 | 0 | 0 | 11 | 0 | 0 | 20 |
| ED-124 | 0 | 0 | 0 | 0 | 0 | 0 | 0 | 0 | 0 | 4 | 0 |
| ED-140 | 0 | 0 | 0 | 6 | 4 | 0 | 0 | 0 | 0 | 0 | 20 |
| ED-143 | 0 | 0 | 0 | 0 | 4 | 0 | 24 | 11 | 2 | 4 | 20 |
| ED-146 | 0 | 0 | 0 | 0 | 0 | 0 | 12 | 11 | 2 | 4 | 20 |
| ED-149 | 0 | 0 | 0 | 0 | 0 | 0 | 0 | 11 | 2 | 0 | 20 |
| ED-156 | 0 | 0 | 0 | 0 | 0 | 0 | 0 | 0 | 0 | 0 | 20 |
| ED-175 | 0 | 0 | 0 | 4 | 4 | 11 | 36 | 12 | 0 | 4 | 20 |
| ICU-002 | 0 | 0 | 0 | 0 | 0 | 0 | 12 | 0 | 0 | 0 | 20 |
| ICU-007 | 0 | 0 | 1 | 4 | 0 | 0 | 12 | 0 | 0 | 0 | 20 |
| ICU-008 | 0 | 0 | 0 | 0 | 0 | 0 | 12 | 0 | 0 | 0 | 20 |
| ICU-009 | 0 | 0 | 0 | 0 | 0 | 0 | 0 | 0 | 2 | 0 | 0 |
| ICU-012 | 0 | 0 | 0 | 0 | 4 | 0 | 0 | 0 | 0 | 0 | 0 |
| ICU-017 | 0 | 0 | 0 | 0 | 0 | 0 | 0 | 0 | 0 | 4 | 0 |
| ICU-019 | 0 | 0 | 0 | 0 | 0 | 0 | 0 | 0 | 0 | 0 | 0 |
| ICU-021 | 0 | 0 | 0 | 0 | 0 | 0 | 0 | 0 | 0 | 0 | 0 |
| ICU-023 | 0 | 0 | 0 | 0 | 0 | 0 | 0 | 0 | 0 | 0 | 0 |
| ICU-024 | 0 | 0 | 0 | 0 | 0 | 0 | 0 | 0 | 0 | 0 | 0 |
| ICU-027 | 0 | 0 | 0 | 0 | 0 | 0 | 12 | 0 | 0 | 4 | 0 |
| ICU-032 | 0 | 0 | 0 | 0 | 0 | 0 | 12 | 11 | 2 | 4 | 0 |
| ICU-035 | 0 | 0 | 0 | 0 | 0 | 0 | 0 | 0 | 0 | 0 | 0 |
| ICU-036 | 0 | 0 | 0 | 0 | 0 | 0 | 0 | 0 | 0 | 0 | 0 |
| ICU-042 | 0 | 0 | 0 | 0 | 4 | 0 | 0 | 0 | 0 | 0 | 20 |
| ICU-046 | 0 | 0 | 0 | 0 | 4 | 0 | 0 | 0 | 0 | 0 | 20 |
| ICU-045 | 0 | 0 | 0 | 0 | 0 | 0 | 0 | 0 | 0 | 0 | 0 |
| ICU-048 | 0 | 0 | 0 | 0 | 4 | 0 | 0 | 0 | 0 | 0 | 20 |
| ICU-051 | 0 | 0 | 0 | 0 | 4 | 0 | 0 | 0 | 0 | 0 | 20 |
| ICU-060 | 0 | 0 | 0 | 0 | 0 | 0 | 0 | 0 | 0 | 0 | 20 |
| ICU-065 | 0 | 0 | 0 | 0 | 0 | 0 | 0 | 0 | 0 | 0 | 20 |
| ICU-069 | 0 | 0 | 0 | 0 | 0 | 3 | 12 | 0 | 2 | 4 | 20 |
| ICU-073 | 0 | 0 | 0 | 0 | 0 | 0 | 0 | 0 | 0 | 0 | 20 |
| ICU-075 | 0 | 0 | 0 | 0 | 0 | 0 | 0 | 0 | 0 | 0 | 20 |
| ICU-077 | 0 | 0 | 0 | 0 | 0 | 0 | 0 | 0 | 0 | 0 | 20 |
| ICU-078 | 0 | 0 | 0 | 0 | 0 | 0 | 0 | 0 | 0 | 0 | 20 |
| ICU-081 | 0 | 0 | 0 | 0 | 4 | 3 | 12 | 0 | 0 | 0 | 20 |
| ICU-082 | 0 | 0 | 0 | 0 | 0 | 0 | 0 | 0 | 0 | 0 | 20 |
| ICU-084 | 0 | 0 | 0 | 0 | 0 | 0 | 24 | 0 | 2 | 0 | 20 |
| ICU-004 | 0 | 0 | 0 | 0 | 0 | 0 | 12 | 0 | 0 | 0 | 20 |
| ICU-005 | 0 | 0 | 0 | 0 | 0 | 0 | 12 | 0 | 0 | 0 | 20 |
| ICU-010 | 0 | 0 | 0 | 0 | 0 | 0 | 0 | 0 | 0 | 0 | 0 |
| ICU-011 | 0 | 0 | 0 | 0 | 0 | 0 | 0 | 0 | 0 | 0 | 0 |
| ICU-018 | 0 | 0 | 0 | 2 | 0 | 0 | 0 | 0 | 0 | 0 | 0 |
| ICU-025 | 0 | 0 | 0 | 0 | 0 | 0 | 0 | 0 | 0 | 0 | 0 |
| ICU-030 | 0 | 0 | 0 | 0 | 0 | 0 | 0 | 0 | 0 | 4 | 0 |
| ICU-031 | 0 | 0 | 0 | 0 | 0 | 0 | 0 | 11 | 0 | 4 | 0 |
| ICU-047 | 0 | 0 | 0 | 0 | 0 | 0 | 0 | 0 | 2 | 0 | 0 |
| ICU-058 | 0 | 0 | 0 | 0 | 0 | 0 | 0 | 0 | 0 | 0 | 20 |
| ICU-068 | 0 | 0 | 0 | 0 | 0 | 0 | 0 | 0 | 0 | 0 | 20 |
| ICU-080 | 0 | 0 | 0 | 6 | 4 | 0 | 0 | 0 | 0 | 0 | 20 |
| ICU-083 | 0 | 0 | 0 | 0 | 0 | 0 | 0 | 0 | 0 | 0 | 20 |

Supplemental Table 3: Summary of missing data

Cell number indicates number of values compared to total expected numbers indicated in column headers. Green indicates all values present, yellow that some values are missing, and red that all values are missing.

| Parameter | | Volunteers  (n=17) | No infection  (n=17) | Mild infection  (n=21) | Moderate infection  (n=37) | Sepsis  (n=42) | p-value |
| --- | --- | --- | --- | --- | --- | --- | --- |
| Age (years) | | 37 (31-44) | 57 (34-65) | 29 (22-36) | 63 (47-82) | 56 (46-74) | <0.0001 |
| Sex (male) n (%) | | 15 (88%) | 9 (53%) | 10 (48%) | 21 (57%) | 26 (62%) | ns |
| Ethnicity n (%) | | | | | | |  |
|  | **Asian** | 6 (35%) | 3 (18%) | 3 (14%) | 1 (3%) | 9 (14%) | 0.0355 |
|  | **Black** | 1 (6%) | 1 (6%) | 1 (5%) | 3 (8%) | 6 (14%) |  |
|  | **Other/Not Stated** | 0 | 3 (18%) | 6 (29%) | 7 (19%) | 4 (10%) |  |
|  | **White** | 10 (59%) | 10 (59%) | 11 (52%) | 26 (70%) | 26 (69%) |  |
| Co-morbidities n (%) | | | | | | |  |
|  | **Diabetes** | - | 0 | 2 (10%) | 8 (22%) | 9 (23%) | ns |
|  | **COPD** | - | 3 (18%) | 0 | 4 (11%) | 7 (18%) | ns |
|  | **Heart failure** | - | 3 (18%) | 1 (5%) | 5 (14%) | 3 (8%) | ns |
|  | **Ischaemic heart disease** | - | 0 | 0 | 3 (8%) | 4 (10%) | ns |
| Source of infection n (%) | | | | | | |  |
|  | **Pulmonary** | - | - | 8 (38%) | 17 (46%) | 16 (42%) | <0.0001 |
|  | **Genitourinary** | - | - | 4 (19%) | 8 (22%) | 2 (5%) |  |
|  | **Gastrointestinal** | - | - | 0 | 1 (3%) | 16 (42%) |  |
|  | **Ear, nose & throat** | - | - | 8 (38%) | 5 (14%) | 0 |  |
|  | **Soft tissue** | - | - | 1 (5%) | 4 (11%) | 0 |  |
|  | **Bacteraemia** | - | - | 0 | 2 (5%) | 3 (8%) |  |
|  | **Other** | - | - | 0 | 0 | 1 (3%) |  |
| No infection group diagnosis n (%) | | | | | | |  |
|  | Heart failure | - | 3 (17%) | - | - | - | N/A |
|  | Migraine | - | 2 (12%) | - | - | - |  |
|  | Non-infective asthma exacerbation | - | 2 (12%) | - | - | - |  |
|  | Non-infective diarrhoea | - | 2 (12%) | - | - | - |  |
|  | Alcohol intoxication | - | 1 (6%) | - | - | - |  |
|  | Fall | - | 1 (6%) | - | - | - |  |
|  | Non-infective COPD exacerbation | - | 1 (6%) | - | - | - |  |
|  | Post-operative joint pain | - | 1 (6%) | - | - | - |  |
|  | Post-vaccine fever | - | 1 (6%) | - | - | - |  |
|  | Rib fractures | - | 1 (6%) | - | - | - |  |
|  | Symptomatic hyponatraemia | - | 1 (6%) | - | - | - |  |
|  | Urinary retention | - | 1 (6%) | - | - | - |  |
| Clinical parameters | | | | | | |  |
|  | **Temperature** | - | 37.0 (36.4-37.9) | 38 (36.8-39.0) | 37.5 (36.7-38.5) | 38.2 (38.0-38.5) | 0.0007 |
|  | **GCS** | - | 15 (15-15) | 15 (15-15) | 15 (15-15) | 14 (11-15) | <0.0001 |
|  | **Respiratory rate** | - | 19 (18-23) | 18 (16-20) | 20 (18-24) | 26 (23-33) | <0.0001 |
|  | **Systolic blood pressure** | - | 135 (123-153) | 131 (116-136) | 134 (118-157) | 93 (88-107) | <0.0001 |
|  | **qSOFA score** | - | 0 (0-1) | 0 (0-0) | 0 (0-1) | 2 (1-3) | <0.0001 |
| Laboratory values | | | | | | |  |
|  | **WBC (x10^6^)** | - | 8.5 (6.8-11.4) | 11.0 (8.1-15.7) | 12.0 (8.0-15.9) | 10.6 (7.2-15.8) | ns |
|  | **Neutrophils (x10^6^)** | - | 6.5 (4.6-8.6) | 8.4 (5.3-12.3) | 10.4 (5.7-14.3) | 8.1 (5.7-11.4) | ns |
|  | **Lymphocytes (x10^6^)** | - | 1.1 (0.7-1.8) | 1.4 (0.7-2.2) | 0.8 (0.5-1.3) | 1.0 (0.7-1.8) | ns |
|  | **Monocytes (x10^6^)** | - | 0.8 (0.5-1.0) | 0.8 (0.5-1.2) | 0.8 (0.5-1.1) | 0.7 (0.4-1.2) | ns |
|  | **CRP (mg/l)** | 1 (1-1) | 6.6 (3.4-14.9) | 27.0 (6.5-49.0) | 91.3 (41.3-149.8) | 173.7 (76.8-247.2) | <0.0001 |
|  | **Lactate (mmol/l)** | - | 1.3 (0.8-2.1) | 1.0 (0.7-1.3) | 1.2 (0.9-1.6) | 1.1 (0.8-2.0) | ns |

Supplemental Table 4: Characteristics of included patients and volunteers.

Abbreviations: COPD: Chronic obstructive pulmonary disease, CRP: C-reactive protein, GCS: Glasgow coma scale, qSOFA: Quick sepsis-related organ failure assessment, n/A: Not applicable, ns: Not significant, WBC: White blood cells.

| Parameter | | Moderate infection  (n=37) | Sepsis  (n=42) |
| --- | --- | --- | --- |
|  | **Positive blood cultures** | 6 (16%) | 13 (32%) |
|  | **Any positive microbiology cultures** | 19 (51%) | 31 (74%) |
| First antibiotic course | | | |
|  | Duration >10 days | 12 (32%) | 28 (68%) |
|  | Escalated to broad spectrum | 6 (16%) | 22 (54%) |
| Second antibiotic course | | 9 (24%) | 15 (37%) |
|  | Median time since cessation of first course | 7 days | 5 days |
| Prolonged course or secondary infection | | **18 (49%)** | **29 (69%)** |

Supplemental Table 5: Subgroup analyses.

# Supplemental Figures

ICU
patients

n = 42

Healthy volunteers

n = 17

ED
patients

n = 74

Volunteers

n = 17

Sepsis

n = 42

Moderate infection

n = 32

No infection

n = 17

Mild infection

n = 25

Total

patients

n = 117

Total

included

n = 134

Supplemental Figure 1: Flow diagram of volunteers and patients included in the study.


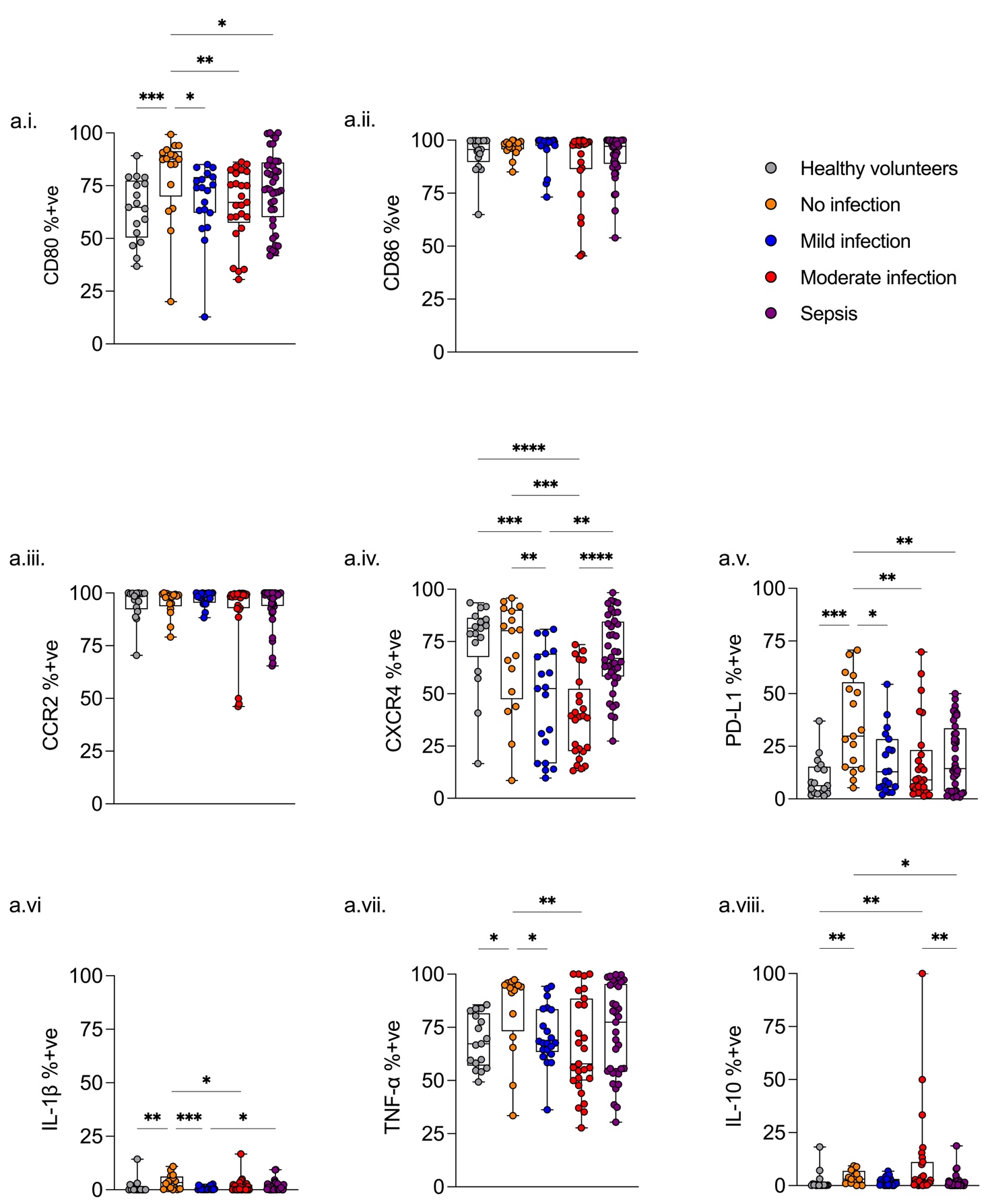


Supplemental Figure 2: Monocyte immunophenotype changes with increasing severity of infection.

Increasing infection severity is associated with alteration in monocyte immunophenotype characterised by expression of (a.i.) CD80, (a.ii.) CD86, (a.iii..) CCR2, (a.iv..) CXCR4, and (a.v.) PD-L1. Intracellular cytokine concentration of (a.vi) IL-1β, (a.vii.) TNF-α, and (a.viii.) IL-10. * p<0.05, ** p<0.01, *** p<0.001, and **** p<0.0001.


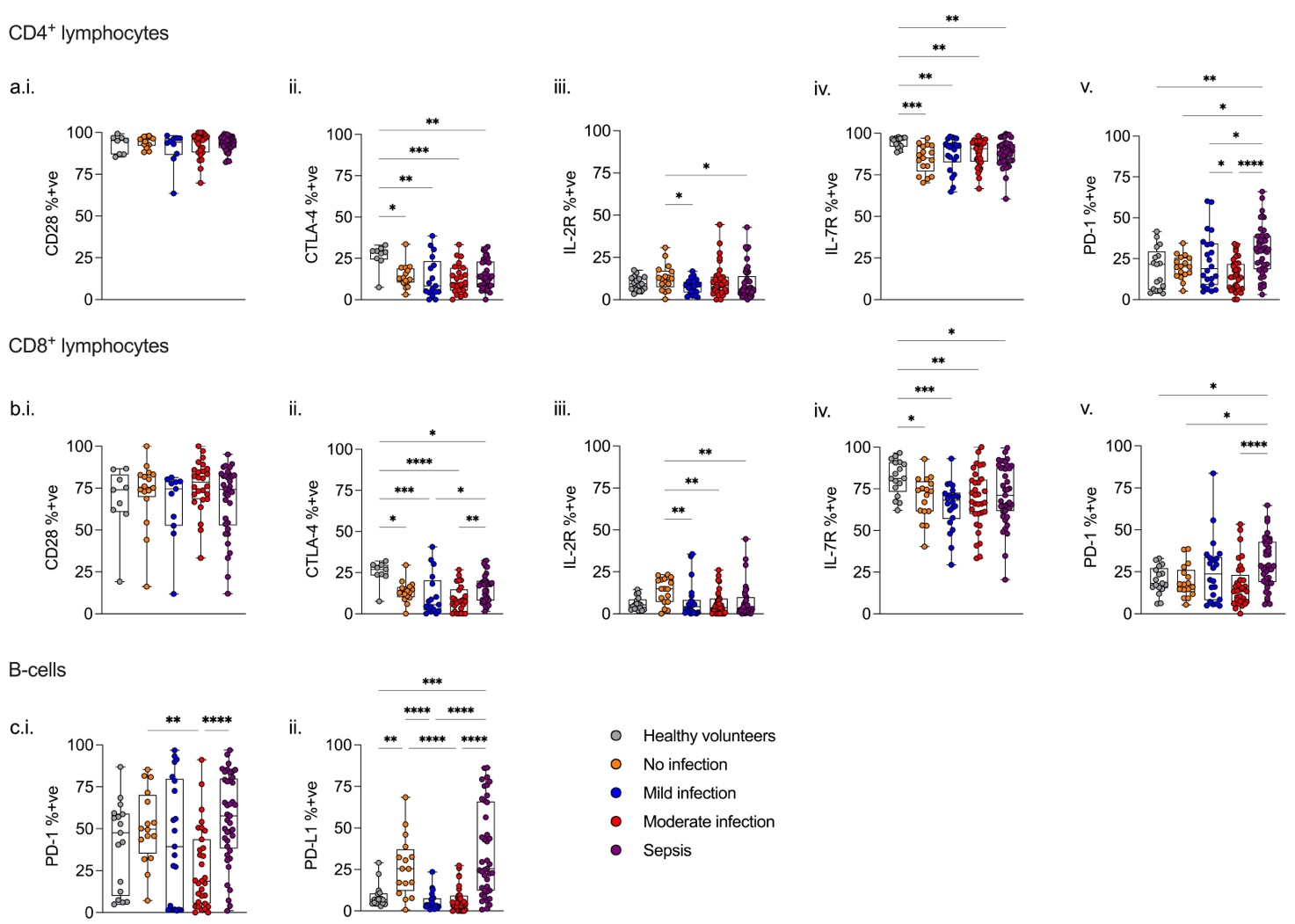


Supplemental Figure 3: Lymphocyte immunophenotype changes with increasing severity of infection.

Increasing infection severity is associated with alteration in immunophenotype characterised by changes in (a.) CD4^+^ and (b.) CD8^+^ lymphocyte expression of (i.) CD28, (ii.) CTLA-4, (iiii.) IL-2R, (iv.) IL-7R, and (v.) PD-1, and in (c.) B-cell (i.) PD-L1, and (ii) PD-1. * p<0.05, ** p<0.01, *** p<0.001, and **** p<0.0001.

Supplemental Figure 4: Changes in serum cytokines, chemokines and growth factors with increasing severity of infection.

In a subset of patients, an exploratory analysis of multiple serum biomarkers was performed including (a.i.) G-CSF, (a.ii.) IL-7, (a.iii.) IL-1RA, (a.iv.) IL-8, (a.v.) IP-10, (b.i.) MCP-1, (b.ii.) IFN-γ, (b.iii.) IL-12p70, (b.iv.) VEGF-A, (b.v.) GM-CSF, (c.i.) IFN-α2a, (c.ii.) IFN-β, (c.iii.) IL-4, (c.iv.) IL-5, (c.v.) IL-9, and (d.i.) MIP-1α. (d.ii.) Correlation between measured cytokines was poor, and (e.) PCA demonstrated minimal separation between groups. * p<0.05, ** p<0.01, *** p<0.001, and **** p<0.0001.

Supplemental Figure 5: Effect of positive microbiological culture result on immunophenotype in moderate and sepsis patient groups.

The immunophenotype of 19 (51%) of patients with moderate infection and 31 (74%) of patients with sepsis who had a positive microbiological culture was compared to those without. Immunophenotype included assessment of (a.-b.) monocyte markers including (a.i.) HLA-DR, (a.ii.) CD86, (a.iii.) CD80, (a.iv.) pHrodo, (a.v.) PD-L1, (a.vi.) CXCR4, (a.vii.) CCR2, (b.i.) IL-1β, (b.ii.) TNF-α, and (b.iii.) IL-10. (c.) CD4^+^ and (d.) CD8^+^ lymphocyte (i.) CD28. (ii.) CTLA-4, (iii.) IL-2R, (iv.) IL-7R, (v.) PD-1, and (vi.) dead. (e.) Cytokines including (i.) CRP, (ii.) PD-1, (iii.) PD-L1, (iv.) IL-1β, (v.) TNF-α, (vi.) IL-6, and (vii.) IL-10. Data compared using Kruskal-Wallis test, * p<0.05, ** p<0.01, *** p<0.001, and **** p<0.0001.

Supplemental Figure 6: Effect of prolonged infection on immunophenotype in moderate and sepsis patient groups.

The immunophenotype of 18 (49%) of patients with moderate infection and 29 (69%) of patients with sepsis who had a prolonged infection was compared to those who did not. Immunophenotype included assessment of (a.-b.) monocyte markers including (a.i.) HLA-DR, (a.ii.) CD86, (a.iii.) CD80, (a.iv.) pHrodo, (a.v.) PD-L1, (a.vi.) CXCR4, (a.vii.) CCR2, (b.i.) IL-1β, (b.ii.) TNF-α, and (b.iii.) IL-10. (c.) CD4^+^ and (d.) CD8^+^ lymphocyte (i.) CD28. (ii.) CTLA-4, (iii.) IL-2R, (iv.) IL-7R, (v.) PD-1, and (vi.) dead. (e.) Cytokines including (i.) CRP, (ii.) PD-1, (iii.) PD-L1, (iv.) IL-1β, (v.) TNF-α, (vi.) IL-6, and (vii.) IL-10. Data compared using Kruskal-Wallis test, * p<0.05, ** p<0.01, *** p<0.001, and **** p<0.0001.
